# Supplementary material for: Genome-Wide Analysis of the Caffeoyl Coenzyme A-O-Methyltransferase (CCoAOMT) Gene Family in Platycodon grandiflorus (Jacq.) A. DC. and the Potential Regulatory Mechanism in Response to Copper Stress
Source: Int J Mol Sci. 2025 May 14;26(10):4709. doi: 10.3390/ijms26104709 (PMC12111519; doi:10.3390/ijms26104709)
Supplement: Supplementary file 1 [file ijms-26-04709-s001.zip › ijms-3516938-supplementary.pdf]

Supplementary File: Amino acid sequences of CCoAOMT in 10 species.

>AtCCoAOMT1

MATTTTEATKTSSTNGEDQKQSQNLRHQEVGHKSLLQSDDLYQYILETSVYPREPESMKE
LREVTAKHPWNIMTTSADEGQFLNMLIKLVNAKNTMEIGVYTGYSLLATALALPEDGKIL
AMDVNRENYELGLPIIEKAGVAHKIDFREGPALPVLDEIVADEKNHGTYDFIFVDADKDX
YINYHKRLIDLVKIGGVIGYDNTLWNGSVVAPPDAPMRKYVRYYRDFVLELNKALAADP
RIEICMLPVGDGITICRRIS

>AtCCoAOMT2

MAKDEAKGLLKSEELYKYILETSVYPREPEVLRELRNITHNHPQAGMATAPDAGQLMGM
LLNLVNARKTIEVGVFTGYSLLLTALTLPEDGKVIAIDMNRDSYEIGLPVIKKAGVEHKIDF
KESEALPALDELLNNKVNEGGFXXAFVDADKLNYWNYHERLIRLIKVGGIIVYDNTLWG
GSVAEPDSSTPEWRIEVKKATLELNKKLSADQRVQISQAALGDGITICRRLY

>AtCCoAOMT3

MSTGLALNRCSVSVCRTAVTLLNRPTVSVARSLKFSRRLIGNCSIAPADPYVVADDDKYGN
KQVISLTPRLYDYVLSNVREPKILRQLREETSKMRGSQMQVSPDQAQLLAMLVQMLAAE
RCIEVGVYTGYSSLAVALVLPESGCLVACERDSNSLEVAKRYYELAGVSHKVNVKQGLAA
ESLKSMIQNGEGASYDFAFVDADKRMYQDYFELLLQLVRVGGVIVMDNVLWHGRVSDP
MVNDAKTISIRNFNKKLMDDKRVSISMVPIGDGMTICRKR

>AtCCoAOMT4

MTTFSTSFLFLLLVFCLIGSLAADDLQHKSGRDVCSGGSDLRTPDIRLNRPTDSVVGNCPTE
ASPLVMADDEKYGNKMVISLTPRLYDYVLNNVREHEILKQLREETAISQIQVSPDQAQLLA
MLVEILGAKRCIEVGVYTGYSSLAVALVLPESGRLVACDKDANALEVAKRYYELAGVSHK
VTVKHGLAAESLMSMIQNGEESSYDFAFLDADKAMYQEYFESLLRLVRVGGVIVIDNVL
WHGWVADSTVNDERTISLRNFNKKLMDDQRVSISMVSIGDGMTICRKR

>AtCCoAOMT5

MDGRLPDKGILKSEALKQYIMETTAYPREHELLKELREATIQRYGNLSEMGVPVDESLFLS
MLVKIINAKNTIEIGVFTGYSLFTVALALPEDGRITAIDIDQAGYNLGLEFMKKAGVDHKIN
FIQSDAVRGLDQLLNGKQEYDFAFVDADKTNYVYFLEKLLKLVKVGGIIAFDNTLWFGTL
IQKENEVPGHMRAYREALLEFNKILARDPRVEIAQISIGDGLTLCRRLI

>AtCCoAOMT6

MANEIPTKGILKSEALKQYIMETSAYPREHELLKELRKATVQKYGNLSEMEVPVDEGHFL
SMLVKIMNAKNTIEIGVFTGYSLLTTALALPEDGRITAIDIDKEAYEVGLEFIKKAGVDHKI
NFIHSDGLKALDQLVNDKCEFDFAFADADKSSYVNFHERLLKLVKVGGIIAFDNTLWFGFV
AEDEDGVPEHMREYRAALIEFNKKLALDPRVEVSQISIGDGITLCRRLV

>AtCCoAOMT7

MEKLLPPSKLLPPKGILKSDALKKYIFETTAYPREHEQLKKLREATVLKYGNLSEMEVPVD
EGHFLSMLLKIMNAKKTIELGVFTGYSLLTTALALPHDGHVTGIDIDKEAYEMGLEFIKNA
GVHHKINFIHSDCLQALDNMLSENPKPEFDFAFVDADKPNYANMHERLMKLVKVGGVIA
FDNTLWSGFVAEKEENVPVHMRVNRKAFLDLNKRLAADPHVEVSQVSIGDGVTLCRRLV

>BnCCoAOMT1

TSVYPREPESMKELREITAKHPWNLMTTSADEGQFLSMLLKLINAKNTMEIGVFTGYSLL
ATALALPEDGKILAMDINRENYELGLPVIEKAGLAHKIDFREGPALPALDQMLEDGKYHG
SFDFIFVDADKDNYLNYHKRLIELVKVGGVIGYDNTLWNGSV

>BnCCoAOMT2

TSVYPREPESMKELREITAKHPWNLMTTSADEGQFLSMLLKLINAKNTMEIGVFTGYSLL
ANGLALPEDGKILAMDINRENYELGLPVIEKAGLAHKIDFREGPALPALDQMIEDGKYHG
SFDFIFVDADKDNYLNYHKRLIELVKVGGVIGYDNTLWNGSV
>BnCCoAOMT3
MANNGEGKQNEVSRHQEVGHKSLLQSDALYQYILETSVYPREPESMKELREITAKHPWN
LMTTSADEGQFLSMLLKLINAKNTMEIGVFTGYSLLATALALPEDGKILAMDINRENYEL
GLPVIEKAGLAHKIDFREGPALPALDQMIEDGKYHGSFDFIFVDADKDNYLNYHKRLIELV
KVGGVIGYDNTLWNGSVVAPPDAPLRKYVRYYRDFVLELNKALAADPRIEICMLPVGDGI
PICRRVS
>BnCCoAOMT4
TSVYPREPESMKELREITAKHPWNLMTTSADEGQFLSMLLKLINAKNTMEIGVFTGYSLL
ATALALPEDGKILAMDINRENYELGLPVIEKAGLAHKIDFREGPALPALDQMIEDGKYHGS
FDFIFVDADKDNYLNYHKRLIELVKVGGVIGYDNTLWNGSV
>Cc.CCoAOMT1
MAPTQAEQQTQASRHQEVGHKSLLQSDKLYQYILETSVYPREPEAMKELRELTAKHPWN
LMTTSADEGQFLNMLLKLINAKNTMEIGVYTGYSLLATALALPEDGKILAMDINRENYEL
GLPVIQKAGVAHKIDFKEGPALPVLDQMIEAGTYHGTFDFIFVDADKDNYINYHKRLIELV
KVGGVIGYDNTLWNGSVVAPPDAPLRKYVLYYRDFVLELNKALAADPRIEICQLPVGDGI
TLCRRIK
>Cc.CCoAOMT2
MATNTQEQQTQAGRHQEVGHKSLLQSDALYQYILETSVYPREPEPMKELRELTAKHPWN
LMTTSADEGQFLNMLLKLINAKNTMEIGVYTGYSLLATALALPDDGKILAMDINRENYEL
GLPVIQKAGVAHKIEFKEGPALPVLDKLVEDEKNHGSYDFIFVDADKDNYINYHKRLIDLV
KVGGLIGYDNTLWNGSVVAPPDAPLRKYVRYYRDFVLELNKALAADPRIEICMLPVGDGI
TLCRRIK
>Cc.CCoAOMT3
MLHRSFKPAKCKTALKLAIPRLKLMKNKREAQVKQLRRELAQLLESGQDQTARIRVEHV
VREEKTVAAYNLLEIYCELIVARMPIIESQKNCPIDLKEAIASVVFASARCGEIPELKDVSKH
FTAKYGKEFTSAALELRPNCGVGRMLVEKLSASAPDGPTKLKILTAIAEEHNIKWDPESFG
AKEAKVYEDLLNLPNTVKEATKIADPPKAQASTSHYEQRTPINQVPTHDKGPPNVQAPKH
MEKNDAPASVYGHSSGSPPYAKNFGNSNSSASNKMSSGTYPPNSKPYGTEHQEMEFRNS
YSGNESAFSSPRQHWNMEFKDATAAAQAAAESAELASMAARAAAELSSRENITRQNSTE
SRMSSAHGMRNDEPHQYTASASQNEHLARRPVAQGRNSQNYGDTDRKELHNRAGQAEN
MYSNIVMSADKSTHGSFKSTAASSIERPSVNNQIGDAYSQRNSSEGRQVEQFSEVTTKRSS
GKNGMQFLSEVHGSKNVDNHEVRVREQSSYSSSHSQLNTSTDDHDVVSNLKWQSSDYD
ERNSSKTRMQFVNELHDIKNSEIADYQEATIRKQSSYSSSHSSSSAFADDHDVVSNLNRQN
SGNNSGEESFPFNDKGSHHRSTKETTDSYDNPSAVFDNYGSDNGGCNFDLEEEHKVHEYS
MDFLSPGRKSPTHPFTSTNSWRIGQTVDSPEKSISQSHIFSEKQSTPVFDESSTSSAVASQRD
DLPAAFDDYGPSSESEEEVEKSKFDRSGDTSIGSDKQNNDFHQSKTSISTPQLAEGIEGTEP
FKDFSMEESKELNLRNLTGGIRNKNKLPPYSRVPQSSTIHSEEATNFTSTRTKQSSTPTAVEA
SVSSGSYNQEPYSRKGSVEVNRKLSTRASRQIQQDSDSSDDDSEEEEIQPYTSTEDQHDKM
PSFEENKVSNLRAPIPYFGSGNSDSDQELPKTSPNSRLNTGLSRRTKASPSNSRRSSNLKTT
VSSEPKVFSDYGGEKYPSLRSSNANEALPRTRPQKKDSDYWESNQQSRLAAQATTKLVSE
TKKSSFDGPPDYGREKYSSLRNSNADEASPRTQPQKKDSDHWESNQQSRLAARTTTKLVS

ETKKSSFDGPPDYGREKYSSLRNSNADEAQPRSQPQKQDSDHWESNQQSRLAARSTNKL
VSETKKSSFDGPPVSSQMEQQAPTSVPKVIASEESMENTSDKQLDKPTKGLLQSTDLYEYI
LETSVYPREAELLKELRAATANHPECIISTAPDAGQLMGMLLKLVNAKKTIEIGVYTGYSL
LLTALSIPSDGQIIAIDINSETYEIGLPIIRKAGVENKINFIESQALPVLDKLLKDKENEGSFDF
AFVDADKNNYWNYHERLLKLVKVGGVIIYDNTLWIGTVARPEEEVSEDKREWRRLRAM
DNEKKESTSFSKGLLQNEELYRYILETSVYPHESEYLKELRDITATHPWSIMATAPDAGQLI
AMLLNLINAKKTIEVGVFTGYSLLLTALTIPEDGKILAIDLNREAYEIGLPVIRRAGVENKID
FRESAALPVLDQLLEDPGNENAFDFAFIDADKINYWNYHERLMKLVKVGGIVAYDNTLW
GGTVAIPEECTPEGVREGRQRTLDFNKLLAADSHVQILLAPLGDGITICRRLH
>Cc.CCoAOMT4
MANVDNISPKCILKSDVLLEYILNTSAYPREHEQLKGLRETTAEKYPNESLMNVPVDEGQF
LSMLLKLMNAKKTLEIGVFTGYSLLATALALPNDGQITAIDPQREFYELGLPFIKKAGMEH
KINFIESEALKVLTEMSNNEKDKPEFDFIFVDADKPNYMKYHEYLKKLVKVGGVIAYDNT
LWFGFLVQEEAQVPEPARPSRKALLELNMSLASDPSMEVCQISIGDGVTLCRRIC
>Cc.CCoAOMT5
MTDWSKKTILRNDALVQYILQTSAFPKEHEQLKELREASAQKYKELSVMNVPADEAQFLS
MLLKLINAKKTLEVGVFTGYSLLATALALPQDGKGEEGSFDFIFVDAYKSDYLKFHELTLK
LVKIGGIIAYDNTLWYGSVAESEKEVTEDLIKRSVMNVPADEGQFLSMLLKMMNAKKTIEI
GVFTGYSLLTTALALPEDGKIIAIDPDKEAYEVGLPFIKKAGIEHKINFIPSDAFLVLNDLINS
GEEGTFDFIFVDASKNDYLKFHELTVKLVKIGGIIAYDNTLFMGSVGKSEEEIKEEPMRQLR
NSVMEFNSFISADPLPRVESSLLSIGDGLTLCRQAVSSLNHKSLIVQPSVLEYILEKNAYPKE
HEQLKKLREVTAEKYEKKSIMNVPADEAQLLSMLLKVMNAKKTMEIGVFTGYSLLATAL
ALPQDAQITAIDLDKEAYETGLPFIKEAGVDHKINFINSDAFLVLDDLINGGDDEGKFDFIF
VDAQKKDYKKFHEQVLKLVKVGGIIAYDNTLWFGSVGYEEEKDWMPEFVWKSREFVLQ
FNSFLATDPRIESSLLSIGDGLTLCKRLY
>Cc.CCoAOMT6
MSAYDYRRMILKSDALQEYIYETSAYPKEHLQLKELREATIEKYQVWSAMSLPVDEAQFL
SLLVKIMNAKKTMEIGVFTGYSLLTTALALPEDGQILAIDPDKEAYEFGLPYLKKAGVEHK
INFVPSDAISYLNGLVNSSEEGSFDFIFVDAFKDQCLEFHEIALKFVKIGGTIGYDNTLWYD
SVAQPEEEVTDEHIRSYRNFVVEFNDFVAADPRVESSIISIGDGSKTLTILDFIRTTAVCRPWK
VCLKDHKPKFPVYLMLAEKEDEDTNNGMRIEEYEDIDEGDGDGVVETKTWIITEGFEIFK
LDTQCRIWEKILSLGDRSLFPGNCCTFSVLAADYPNCNSNYVMNDDSHSWYRKGPGDYD
IGIYNCDNKEVLQLPVSDDKQRFRLNSPHLFGSI
>Cc.CCoAOMT8
MHSAPPLSLPRMATSFTISRCSASACQRAVVFLSRTQRCHSFSPRTAKFRFLKLNHLTRNCS
SSPKAPFIVADDEKYGNKQVISITPRLYDYILGNVREPQILRQLREETANMRGSQMQVSPD
QAQLLAMLVQILGAERCIELGVYTGYSSLAIALALPESGCLVACERDARSLEVAKKYYALA
GVSDKIIVKHGLAADALKSMILNGEACSYDFAFVDAEKKMNQEYFELLLQLVRVGGVIVI
DNVLWHGKVADPLVNDAKTVSIRNFNRSLMEDNRVSISMMLKMLASETCL
>Cc.CCoAOMT9
MSSDSSQRVFQLKLDPLTGNSEWVVIEDNDELQETSNEPLLATTSYLDMLNDSYRNKAFR
LAIEKTVTKPCHVLDIGAGTGLLSMMAARAMGLNGKVTACEAYLPMAKLMRKVLHRNG
MTKNINLINKRSDELEVGIDIPSRADVLVSEILDSELLGEGLIPTLQHAHDKLLVENPLTVPY
RAVTYGQLVESSYLWKLHDLSGNEAEGSDSIHLVPTGLDTIIDVKSRQYPMHCDAIRKE

IKLLSEPFKIFEFDFWKRPDSHGETEVQIKAIGDGNIHAVVSWWILQLDREGTVFYSTAPR
WINSTANVGDRNWCDHWKQCVWLLPGKGMPVSKEEEVLLRATHTETSVSYNLNVQVPQ
TDRRQHDHRIGDLQLLLSPERIAAYGDSEWQLSTLAAVRNALQGKVNPLCVVADDSIFLTL
LAANLSKTSHVIALFPGLRGKGAQYVRTVSKANGFSTDHVEVPEQRKACLTMYDTHGKK
IDLLIGEPYYYGNEGMLPWQNLRFWKERTLLDPVLSENALVMPCKGILKACAMSLPDLW
RSRRCLGELEGFEHSIVNTTLGACGELPALKEGPYLPFSIWQCGEIKELSETFTILEFDCSKPI
SSCYGKAQVQFNEHGICHGFVLWMDWVMDPENSIVASTGPDQRYWKQGVKLLAKPIAV
GIHGSQSTSEFGSAVMEAFFDPSNSELIIKHIFS
>CsCCoAOMT1
MATNAEINGQNKENQAGRHQEVGHKSLLQSDALYQYILETSVYPREPEPMKQLRQLTAN
HPWNIMTTSADEGQFLNMLLKLINAKNTMEIGVYTGYSLLATALALPHDGKILAMDINRE
NYELGLPIIQKAGVAHKIDFREGPALPVLDQMIEDTEYHGSFDFIFVDADKDNYLNYHKRL
IDLVKVGGLIGYDNTLWNGAVVAPPDAPLRKYVRYYKDFVLELNKALAVDPRIEICQLPV
GDGITLCRRIS*
>CsCCoAOMT2
MIISTIQIINRKKYNSFSFYDVNSKVKLGKAMADTIVLKTILQSEALQKYILDTNAYPREHE
QLKWLRDATFKKYGNRAELSVPPDEGLFLSMLLKLMNAKKTLEIGVFTGYSLLTTALALP
HDGQIVAIDPNREAFEVGLPFIQKAGVEHKINFIESDAISVLNEMLSDEGKLKMEFDFVFVD
ADKPNNINYHEQAIKLVKVGGVIAYDNTLYRGSVVNNEEEVPERFRANQKPIIELNKHLAS
DPRIEIAQISIGDGVTLCRRIL*
>CsCCoAOMT3
MDWSPVFAKNAYLDTLKLCSKHEEQCDSCSTSEPESKEFISALAAGMNAKFIVEVTPTASP
STIALAAAARQTGGKLVCILPEPRLGESQKVIDDSGLNDMVEFKTGDPAELLSSYENIDFSL
VDCKTDNYTNLLEMLDVNPTRSMVVADNLVEGRTGLGGHVKGMENKGKVRSTKHPIGK
GMEVTMIGKLGDEFGKKSHSKGCHSRGRGGGGGGGSGKRSGKSEWVIKVDENSGEEHF
FRMPKSL*
>CsCCoAOMT4
MAASLLPRSTYPRLIFAPLFSLQRDLLLPSFDRLVHVSSPIAAFNRRFRAQNSNNSADATVV
AGDENYGNKRVISVTPRLYDYLLSNVREPEILKELREETATRRGSQMQVSPDQAQLLAML
VQILGAEKCIEVGVYTGYSSLAIALVLPESGRLVACERDMKSLEVAKRYYDRAGVSHKVD
VKHGMAADALKSMIQKGESCSYDFAFVDAEKRMYHEYFELLLQLVRVGGLIVIDNVLWH
GKVANPLVNDAKTVSIRNFNRSVMEDKRVSISMVPIGDGMTICRKL*
>CsCCoAOMT5
MATNGEGEQNLRHQEVGHKSLLQSDALYQYILETSVYPREPEAMKELREVTAKHPWNIM
TTSADEGQFLNMLLKLINAKNTMEIGVYTGYSLLATALALPDDGKILAMDINRDNFEIGLP
IIEKAGVAHKIDFREGPALPALDKMIEDGKHHGSFDFIFVDADKDNYINYHKRLIDLVKVG
GLIGYDNTLWNGSVVAPPDAPMRKYVRYYRDFVLELNKALAADPRIEICMLPVGDGITLC
RRVC*
>CsCCoAOMT6
MGDLKMKNILRSEALTKYILETSAYPREHQLLKELREATVNKYKERSLMSVTVDEGQLIS
MLLKIMNAKKTVEIGVFTGYSLLTTVLALPHDGKVIAIDPRREAYEVGLPFIKKVGVEHKI
NYIQSDGMTVLNNLLTNEKQEGSFDFAFVDADKENYMNYHELLLKLVKIGGIIAYDNTLW
NGSVVPSKYEEEAEDSARDALRKLNSFLASDSRVDLSHVSIADGLTLCRRLS*
>CsCCoAOMT7

MADNIVLKTILKSEALQKYILDTSAYPREHEQLKRLRDATFKKYGDRAEMSVPPDEGMFL
SMLLKLMNAKKTLEIGVFTGYSLLTTALALPHDGQIVAIDPNREAFEVGLPCIQKAGVEHK
INFIESDATSILNEMLSDEGKLKMEFDFVFVDADKPNSINYHEQAIKLMKVGGVIAYDNTL
WRGSVVSKEEEVPERFRANQKPIIELNKRLASDPRVEIAQISIGDGVTLCRRIL*
>CsCCoAOMT8
MASVAVGARSSVTTAATGPGVTMKYIFDTNVYPREHEQLKRIRDATFKKYGYRAELSVPP
DEGLFLSMLLKLMNAKKTLEIGVFTGYSLLTTALALPHDGQIVAIDPNREAFEVGLPFIQK
AGVEHKINFIESDAISVLNEMLSDEGKLKMEFDFVFVDADKPNYINYHEQAIKLVKVGGVI
AYDNTLWYGSVVSNEEEVPERLRASQKPIIELNKYLASDPRIEITQISIGDGVTLCRRIL*
>CsCCoAOMT9
MANRVPEPNPVVLQSDQLYQYILETSVYPRESEHLKQIRDATATHPAAFFGTAPDAGQLMA
MLLKLVNAKKTIEVGVFTGYSLLLTALTIPDDGQIVAIDINREAYEIGLPIIQKAGVEHKIDFI
QSPALPVLDKLLEDKENEGAFDFAFVDADKGNYLNYHERLMKLVKDGGLLVYDNTLWG
GTVAKPEESVPVYKRQWRKATIEFSKSITADHRVEISHVPSGDGVIICRRIC*
>CsCCoAOMT10
MEQTTKKVTSPTKGLLQSDELYQYILETSVYPREPELLKELREVTASHPRSMMGTAPDAG
QLMAMLLKLVNAKNTIEIGVFTGYSLLLTALSIPDDGKIIAIDLNQETYEIGLPVIRKAGVE
HKINFIVSEALPVLDKLLENNENEGSFDFAFVDADKVNYLNYHERLMKLVKIGGIVVYDN
TLWGGTVAMPEESVPEGMKHGRHFTIELNKSLASDPRILMSHAPLGDGITICKRIQ*
>GmCCoAOMT1
MTLIKELEQQPNQIAGHKELAHKSLLQSDALYQYILETSVYPREHESLKELRELTEKHPWN
LMATPPDEGQLLGMLLKLINAKNTMEIGVFTGYSLLSTALALPSDGKAGVAHKIDFREGPA
LPLLDQLIKDEKNKGAFDFIYVDADKDNYLNYHKRVIELVKVGGLVGYDNTLWNGSVVA
PPDAPLMDYVKYYRDFVMELNKALALDSRVEICQLPVGDGITLCRRII
>GmCCoAOMT2
MSSNPVILQSENLTKYILETSVYPREEETLKELRNATASHPWGFMGAAPDAGQLMTLLLK
LLNAKKTIEVGVFTGYSLLLTALTIPDDGKIIALDPDREAYEIGLPFIKKAGVEHKIDFIESPA
LPVLDKLIEDPSNKESFDFAFVDADKDNYWNYHERLLKLVKIGGLIIYDNTLWGGTVAWP
EEDVPAPKRKFRQAALAFNKAIADDSCVEISAVSIGDGFTICRRAH
>GmCCoAOMT3
MDNISKPEVILQSEGLLKYILETGVYPREAEILKELRNATAEHPLGFMGAAPDAGQLMAM
LLKLLNAKKTIEVGVFTGYSLLLTALTIPNDGKIIAMDPDRKAYEIGLPFIKKAGVEHKIDFI
ECPALPVLDKLLEEPANEGSFDFAFIDADKNNYWNYHERLIKLVKIGGLVAYDNTLWGGTV
ALPEKAVSEPKREWRRLSLAFNKAISKDCRVQIAFLSIGDGVIICMRVR
>GmCCoAOMT4
MAEQNQNQTTEAGRHQEVGHKSLLQSDALYQYILETSVYPREPESMKELRELTAKHPWNI
MTTSADEGQFLNMLLKLINAKNTMEIGVYTGYSLLATALALPEDGKILAMDINRENYELG
LPVIKKAGVDHKIEFREGPALPVLDEMIKDEKNHGSYDFIFVDADKDNYLNYHKRLIELV
KVGGVIGYDNTLWNGSVVAPPDAPLRKYVRYYRDFVLELNKALAVDPRIEICMLPVGDGI
TICRRIK
>GmCCoAOMT5
MAEEERHCKSKRGLTKHKMSSNPVILQSVNLTKYILETSVYPREEETLKELRKATAGHPW
GFMGAAPDAGQLMTLLLKLLNAKKTIEVGVFTGYSLLLTALTIPDDGKIIALDPDREAYEI
GLPFIKKAGVEHKIDFIESPALPVLDKLLEDPSNKESFDFAFVDADKDNYWNYHERLLKLV

KIGGLIIYDNTLWGGTVAWPEEDVPVPKRKLRQATLAFNKAIADDSRVEISVVSIGDGFTIC
RRAH
>GmCCoAOMT6
MPKPCCSMHKHYYKTNLANPGPDANRSNQTSHRFEPAFSFLISITLLHPPTSSSSNYQLFQ
KGEEKERKQNAQRIIIAMAEQNQNQTTEAGRHQEVGHKSLLQSDALYQYILETSVYPREP
ESMKELRELTAKHPWNIMTTSADEGQFLNMLLKLINAKNTMEIGVYTGYSLLATALALPE
DGKILAMDINRENYELGLPVIKKAGVDHKIEFREGPALPVLDEMVKDEKNHGSYDFIFVD
ADKDNYLNYHKRLIELVKVGGVIGYDNTLWNGSVVAPPDAPLRKYVRYYRDFVLELNKA
LAVDPRIEICMLPVGDGITICRRIK
>GmCCoAOMT7
MENIKDPSIYRNPVILQSEDLTKYILETAVYPREPAPLKELREATNNHPWGFIATLPEAGQL
MTLLLKLLNPKKTIEVGVFTGYSLLLTALNIPHDGKITAIDINRKTYEVGLPVIKKAGVEHK
IDFIESPALPILDKLLEDPANEGSFDFAFIDADKENYVNYHERLIKLVKIGGLLVYDNTLWG
GRVCWPEDKVPPHARSGRDAAIEFNKTITNDSRVEFALTSVGDGLNICRRVAI
>HcCCoAOMT
MATNTQEQQSQAGRHQEVGHKSLLQSDALYQYILETSVYPREPESMKELRELTAKHPWN
LMTTSADEGQFLNMLLKLINAKNTMEIGVYTGYSLLATALALPDDGKILAMDINRENYEL
GLPVIQKAGVAHKIEFKEGPAMPVLDQLVEDEKNHGSYDFIFVDADKDNYINYHERLIKLV
KVGGLIGYDNTLWNGSVVAPPDAPLRKYVRYYRDFVLELNKALAADTRIEICMLPVGDGI
TLCRRVK
>IiCCoAOMT1
MENLIPSKGILKNEALKKYIFETTAYPREHEELKKLREATVHKYGNLSEMEVPVDEGLFLS
MLIKMMNAKNTLELGVFTGYSLLTTALALPDDGRITAIDIDREAYEVGLEFIKNAGVDHKI
NFIQSDGLQALDKMLSENPKPEFDFAFVDADKPNYVNALERLMKLVKVGGIIAFDNTLWF
GFVAEEEESVPEHMRVNRKALMELNERLASDPRIELSQVSIGDGVTLCRRLV*
>IiCCoAOMT2
MLVQILGAEKCIEVGVFTGYSSLSVALASPETGRLVACERDLNSLEVAKKYYKLAGVSNK
VNDAKTVSIRDFNKRLMVDERVSIMESLPHDVVELILERLAVKFLVRLRCVSKKWKSTIDS
RRFQEPHFIRSSKSRDADVLFVSIRD*
>IiCCoAOMT3
MAKDEARASDFSKGLLQSEELYKYILETTVYPREPEALKELRNITHNHPRAKMATAPDAG
QLMEMLLNLVNARKTIEVGVFTGYSLLLTALTLPEDGKVIAIDVNRESYEIGLPVIKKAGV
EHKIDFKESEALPVLDELLNDKDNEGGFDFAFVDADKVNYWNYHERLMRLIKVGGIIVY
DNTLWGGSVAEPDSSTPEWRREGKKATLELNLKLSADQRVQISHAALGDGITICRRLC*
>IiCCoAOMT4
MATTTTEATKTSTNGEDKQSQNLRHQEVGHKSLLQSDDLYQYILETSVYPREPESMKELR
EVTAKHPWNIMTTSADEGQFLNMLIKLINAKNTMEIGVYTGYSLLATALALPEDGKILAM
DVNRENYELGLPIIEKAGVAHKIDFREGPALPVLDQLVADEKNHGTYDFIFVDADKDNYIN
YHKRLIDLVKVGGVIGYDNTLWNGSVVAAPDAPMRKYVRYYRDFVLELNKALAADPRIE
ICMLPVGDGITICRRIN*
>IiCCoAOMT5
MDHALPNKGILKSEALRQYIMETTAYPREPELLKELRYATIERYGNLSEMGIPIDEGLFLSMI
VKMMNAKNTLEIGVFTGYSLFTVALALPEDGRITAIDMDKAGYNMGLEFMTKAGVDHKI
NFIKSDALEALDQLLKGEKQEFDFAFVDADKRNYVNFLERLLKLVRVGGIIAFDNTLWFG

TVAEEENEVPHHLKLYREAIMQFNKKLATDSRVEISQVSIGDGSNLDASCHIFTTYGEISRIS
KSLFFSHFKIPTTLTRFQVDR*
>IiCCoAOMT6
MANEIPPKGILKSEALLQYILETSAYPREHELLKELRKVTAQRYGNLSEMVVPVDEGIFLS
MLIKIMNAKNTLELGVFTGYSLLATALALPEDGRITAIDIDKETYQVGLEFLKKAGVDHKI
NFIHSEGMKALDQLVNDKQEFDFIFADGDKPNYVNFHEKFLKLVKVGGIIAFDNTLWFSF
VVEDEERVPEFMRESRVALLEFNKKLALDPRVEISQISVGDGVTLCRRLV*
>IiCCoAOMT7
MANEIPSKGILKSEALKQYILETSAYPREHELLKELRKATVHKYGNLSEMEVPVDEGHFLS
MLLKIMNAKNTLELGVFTGYSLLTTALALPEDGRITAIDIDKEAYEVGLEFIKKAGVDHKI
NFIHSDGIKALDQLVNDNEEFDFAFADADKSNYVNFLERLLKLVKVGGIIAFDNTLWFGFV
AEDEEGVPEHMREYREALIEFNKKLALDTRVEVSQISIGDGVTLCRRLI*
>IiCCoAOMT8
MATGLAMARCSISVCRTADVLLNRSTVSVVRNLKFSRRLIGNCSMAATPFVVADDEKYGN
KQVISLTPRLYDYVLSNVREPEILRKLREETAKMRGSQMQVSPDQAQLLAMLVQILGAER
CIEVGVYTGYSSLAVALALPESGRLVACERDSNSLEVAKRYYELAGVSHKVIVKHGLAAES
LKSMIQNGEGSSYDFAFVDADKRMYQDYFELLLQLVRVGGVIVMDNVLWHGRVADPMV
NDAKTISIRNFNKKLVDDERISISMVPIGDGMTICRKR*
>OsCCoAOMT1
MAATGAGEGKETAAVAGGGGGGSLHSKTLLKSEPLYQYVLESTVFPREPDCLRELRLATA
NHPMAVMAASPDQVQLFGLLIELISAKNAIEVGVFTGYSLLATALALPDDGKIVAIDVSRES
YDEVGAPVIDKAGVAHKVDFRVGLAMPVLDELVAEEGNKGRFDFAFVDADKVNFLGYH
ERLLQLVRVGGLIAYDNTLWGGSVAAPPAAADEAVPSGRDRSLAALAREFNAAIAADRRV
KPCQLAIADGVMLCRRVA
>OsCCoAOMT2
MPLLVTLLPVYCTAHSRRLKRTTPASRVSSTAMAAANGDASHGANGGIQIQSKEMKTAIHS
NDSPKTLLKSESLHEYMLNTMVYPRENEFMRELRLITSEHTYGFMSSPPEEGQLLSLLLNL
TGAKNTIEVGVFTGCSVLATALAIPDDGKVVAIDVSREYFDLGLPVIKKAGVAHKVDFREG
AAMPILDNLLANEENEGKFDFAFVDADKGNYGEYHERLLRLVRAGGVLAYDNTLWGGS
VALEDDSVLEEFDQDIRRSIVAFNAKIAGDPRVEAVQLPVSDGITLCRRLV
>OsCCoAOMT3
MWGLVDAKLIRVSTAMHRFASASSLPPPAPATAAAAAAQAAALRFGSAATTRVPRALALT
ASTCPWHRRHLCSSSSSSSSAAAAAATAAAVEEARQGRKQLGATTQLYEYLLANVREHPV
LKELREETAAMRGSQMQVSPAQAQLLAMLAQILGAQRCIEVGVYTGYSSLAVALALPESG
RLVACERDERCLEVAKKYYQRSGVAHKVDVKHALAADSLKLLIDGGEVNSYDFAFVDAD
KRMYEEYYELLLKLVRVGGLIVIDNVLWYGRVADPLVNDRKTISIRNFNKKLLEDNRVSIS
MVPIGDGMTICRKLVDT
>OsCCoAOMT4
MAEAASAAAAATTEQANGSSGGEQKTRHSEVGHKSLLKSDDLYQYILETSVYPREHECM
KELREVTANHPWNLMTTSADEGQFLNLLLKLIGAKKTMEIGVYTGYSLLATALAIPDDGT
ILAMDINRENYELGLPSIEKAGVAHKIDFREGPALPVLDQLVEEEGNHGSFDFVFVDADKD
NYLNYHERLMKLVKVGGLVGYDNTLWNGSVVLPADAPMRKYIRYYRDFVLELNKALAA
DHRVEICQLPVGDGITLCRRVK
>OsCCoAOMT5

MATYRPGSNTLLKSDSILEYVLDTTVYPREHERLRELRLITQNHPKSFMGSSPDQMQFFSV
LLKMIGARNAVEVGVFTGYSLLATALALPDDGKVVAIDVSREYYELGRPVIEDAGVAHKV
DFRHGDGLAVLDQLLAGGEGKFDFAYADADKEQYRGYHERLVRLLRVGGVVAYDNTLW
GGSVAMPRDTPGSSAYDRVVRDYMVGFNAMVAADDRVEACLLPVADGVTLCRRLK
>OsCCoAOMT6
MTTGNGDAPVIKNAHSDIDSTNKTLLKSDALYKYVLDTTVLPREPECMRDLRLITDKHQ
WGFMQSSADEAQLLGMLLKMAGAKRTIEVGVFTGYSLLATALALPEDGKVVAIDPDRES
YEIGRPFLEKAGVAHKVDFREGKGLEKLDELLAEEAAAGREAAFDFAFVDADKPNYVKY
HEQLLQLVRVGGHIVYDNTLWAGTVALPPDTPLSDLDRRFSVAIRDLNSRLAADPRIDVCQ
LAIADGITICRRLV
>TcCCoAOMT1
MDNEKKESTSFSKGLLQSEELYQYILGTSVYPRESEHLKELRDITATHPRAVMATAPDAGQ
LIALLLKLINAKRTIEVGVFTGYSLLLTALTIPEDGKIVAIDMNREAYEIGLPIIRRAGVDNKI
DFIESEALPVLDQLLEDPGNENGFDFAFIDADKINYWNYHERLMKLVKVGGIVVYDNTLW
AGTVALSEQSTPEAMREGRQRTLDVNKLLAADSRVQISLAPLGDGITICRRIL
>TcCCoAOMT2
MADTPMKMILRTEALRKYIYETSAYPKEHEQLKELRETTVEKYQKRSFMSTPVDEAQFLS
MLVKIMNAKKTMEIGVFTGYSLLTTALALPQDGKILAIDRDKEAYEFGLPYIKKAGIEHKI
NFVASDAISALNDLVNSGEEGTFDFIFVDALKSEYLKYHELTMKFVKIGGVIAYDNTLWSG
SVAQAEDQVEMPGVSISELLNLFQELRIVLLPDESE
>TcCCoAOMT3
MLYLFPFYNLLVGFNLPFNGEDIFQEFRSMADTPMKMILRTEALRKYIYETSAYPKEHEQL
KELRETTVEKYQKRSFMSTPVDEAQFLSMLVKIMNAKKTMEIGVFTGYSLLTTALALPQD
GKILAIDRDKEAYEFGLPYIKKAGIEHKINFVASDAISALNDLVNSGEEGTFDFIFVDALKSE
YLKYHELTMKFVKIGGVIAYDNTLWSGSVAQAEDQVEMPGIRSFRKFVIEFNSFIAADPRV
ESSLLSIGDGVTLCRRLY
>TcCCoAOMT4
MANMNTSSKGLLKSEALKKYILNTSAYPREHEQLKGIRDATAQKYPNLCAMGVPVDEGQ
FLSMLLKLMNAKRTMEIGVFTGYSLLATALALPDDGKITAIDTSWEFFEVGLPFIKKAGME
HKINFIESDAMKVLNEMSSNDKQKPEFDFVFVDADKTSYMKYHEHIKKLVKIGGVVAYD
NTLWFGFLAQEEAEVPESARASRKAMLNFNVNLASDPCMEVSQVSIGDGVTLCRRIS
>TcCCoAOMT5
MAVSSLKYKSLIVHRPILEYILEKNAYPKEHEQLKELREATAEKYGKKSIMNVPADEAQFL
SMLLKVMNAKKTLEIGVFTGYSLLATALALPDDGQITAIDLDKEAYETGLPFIKKAGVEHK
INFVHSDAFLVLNDLINGENEETLDFIFVDAEKKDYMKLHEQVLKLVKVGGIIAYDNTLWF
GSVAYEEEKDEMPEFVWGTREYVVQFNSFLAADPRIELSLLSIGDGVTLCRRLH
>TcCCoAOMT6
MATNTQEQQSQAGRHQEVGHKSLLQSDALYQYILETSVYPREPEPMKELRELTAKHPWN
LMTTSADEGQFLNMLLKLINAKNTMEIGVYTGYSLLATALALPDDGKILAMDINRENYEL
GLPVIQKAGVAHKIDFKEGPAMPALDQLVEDEKNHGSFDFVFVDADKDNYINYHKRLIEL
VKVGGLIGYDNTLWNGSVVAPPDAPLRKYVRYYRDFVLELNKALAADPRIEICMLPVGD
GITLCRRVK
>TcCCoAOMT7
MAPTQEGQQNQAGRHQEVGHKSLLQSDNLYQYILETSVYPREPEPMKELRELTAKHPWN

IMTTSADEGQFLNMLLKLINAKNTMEIGVYTGYSLLATALALPDDGKILAMDINRENYEL
GLPVIQKAGVAHKIDFKEGPSLPVLDQMIEAGKYHGTFDFIFVDADKDNYLNYHKRLIEL
VKVGGVIGYDNTLWNGSVVAPPDAPLRKYVLYYRDFVLELNRALAADPRIEICQLPVGDG
ITLCRRIS

Supplementary Table S1: Primers used for RT-qPCR

Gene	Forward primer（5′-3′）	Reverse primer（5′-3′）
PgGAPDG	CAGGGAGGCTTTTAGTTCAGGT	ATCACATCTACACCCCTCCAGC
PgCCoAOMT1	CGACAACACGCTATGGTACG	AACTCGGGGATCTGAAGCAA
PgCCoAOMT2	CCACCAGATGCACCAATGAG	CGGGGAGCATACAGATTTCG
PgCCoAOMT3	ATCCCGTGTCGATGATCTCC	CTAGTCGACTTCCACGCTCT
PgCCoAOMT4	TCAGATGGTGGCAGGAAAGT	CGTTTTCCTCGCGTCTTGAA
PgCCoAOMT5	CCCTTGCTCTCCCTGATGAT	TCAAGAACAGGCAAAGCAGG
